# Effects of age of onset on clinical characteristics in schizophrenia spectrum disorders

**DOI:** 10.1186/1471-244X-10-63

**Published:** 2010-08-18

**Authors:** Yu-Chen Kao, Yia-Ping Liu

**Affiliations:** 1 Department of Psychiatry, Songshan Armed Forces General Hospital, Taipei, Taiwan; 2 Department of Physiology and Biophysics, National Defense Medical Center, No.161, Section 6, Min-Chuan East Road, Taipei 114, Taiwan

## Abstract

**Background:**

Over the last few decades, research regarding the age of onset of schizophrenia and its relationship with other clinical variables has been incorporated into clinical practices. However, reports of potential differences in demographic and clinical characteristics between early- and adult-onset schizophrenia spectrum disorders have been controversial. Thus, this study aims to assess differences in demographic and clinical characteristics correlated with age of illness onset in schizophrenia spectrum disorders.

**Methods:**

Data were collected from 104 patients with schizophrenia and schizoaffective disorder. Diagnosis was made via structured clinical interviews. Assessments of psychiatric symptoms and social and global functioning were completed. The effect of age of onset on demographic and clinical variables was examined using correlation analyses and binary logistic regression models. We chose 17 years of age as the cut-off for early-onset schizophrenia spectrum disorders based on a recent clinical consensus. We further investigated differences in the severity of psychopathology and other clinical variables between the early- and adult-onset groups.

**Results:**

The binary logistic regression analysis showed that age of onset was significantly related to the cognitive component of the Positive and Negative Syndrome Scale (PANSS) (odds ratio, OR = 0.58; 95% confidence interval, CI = 0.872-0.985; p < 0.001) and Barratt Impulsiveness Scale (BIS) score (OR = 0.94; 95% CI = 0.447-0.744; p = 0.015). Patients with early onset of schizophrenia spectrum disorders had significantly greater levels of cognitive impairment and higher impulsivity. There were significant differences between several demographic and clinical variables, including the negative symptom component of the PANSS (p < 0.001), cognitive component of the PANSS (p < 0.001), BIS score (p = 0.05), and psychological domain of quality of life (QOL) (p = 0.05), between patients with early- and adult-onset schizophrenia spectrum disorders, having controlled for the effect of the current age and duration of illness.

**Conclusions:**

Our findings support the hypothesis of an influence of age of onset on illness course in patients with schizophrenia spectrum disorders. This finding may in fact be part of a separate domain worthy of investigation for the development of interventions for early symptoms of schizophrenia.

## Background

Schizophrenia is a complex, chronic, and disabling illness that presents with heterogeneity in its clinical appearance, in patterns of psychopharmacological response and in long-term outcomes [1,2]. While the last few decades of research have given rise to a tremendous wave of interest regarding the natural illness course of schizophrenia, the overarching goal of this research of influencing the prognosis and outcome for schizophrenia patients remains pertinent today. The validity of possible predictors of treatment response and long-term outcome in schizophrenia patients has been a topic of much study in recent years. However, the literature still lacks a clear-cut picture regarding which factors are valuably prognostic in the clinical management of schizophrenia spectrum disorders.

Numerous empirical studies concentrate on the predictive values of the variables detectable at the first episode of schizophrenic illness for the long-term patient outcome. Some practical factors, including sex and age at onset, have been reported as being among the most important determinants of the outcome of schizophrenia [3]. A number of studies have suggested that women in general may have a later age of onset and an overall lower severity of illness, suggesting a protective effect of estrogens [4]. Several negative predictors of outcome in schizophrenic patients have been identified, including male sex [5], early and non-acute onset of syndromes [6], the prevalence of negative symptoms [7,8], the presence of affective symptoms [9], poor pre-morbid functioning [10], and a delay in starting pharmacological treatment [11]. All of these predictors have been associated either with poorer long-term global functioning or with higher rates of relapse or hospitalisation. In particular, patients with schizophrenia with earlier ages of onset are more likely to be males and to have poor pre-morbid adjustment, lower educational achievement, more evidence of structural brain abnormalities, more prominent negative symptoms, more cognitive impairment, and a worse overall outcome [12] as well as a higher likelihood of having relatives with schizophrenia [13,14].

The onset of schizophrenia prior to age 13 is exceedingly rare [15], but an estimated 39% of males and 23% of females with schizophrenia develop the illness by the age of 19 [16]. Patients with early-onset schizophrenia (EOS; onset by age 18) [17], including childhood-onset schizophrenia (COS; onset by age 13) [16] and adolescent-onset schizophrenia (AOS; onset after age 13 and before age 18) show a number of the same neurobiological abnormalities observed in adult-onset schizophrenia, suggesting the involvement of a common neurobiological substrate [17,18]. However, compared to patients with adult-onset schizophrenia, an early onset of schizophrenia appears to be associated with higher rates of pre-morbid abnormalities [17,19], worse cognitive performance [20] and worse functional outcomes [21]. Taken together, these studies indicate that EOS may result in a more severe form of the disorder.

While age of onset and sex have been documented to be fundamental for understanding schizophrenia and bipolar disorder separately [3,22], there is also substantial clinical and neuropsychiatric overlap between these two disorders. We have embodied this overlap in schizoaffective disorder, a diagnostic category with features of both schizophrenia and affective disorders but with poor construct validity [23]. The current diagnostic system has been further called into question by recent studies indicating shared genetic determinants of bipolar disorder and schizophrenia [24]. In spite of these overlaps, there has been little research examining the characteristics and symptoms of psychosis within both schizophrenia and bipolar disorder, which could yield evidence of commonalities as well as differences among patients with psychosis [25].

In this study, we estimated the relationship of age of onset to other clinical characteristics in well-classified patients diagnosed with schizophrenia spectrum disorders. We also compared psychopathology and other clinical variables between schizophrenia and schizoaffective patients with early and adult onset. With regard to the age of onset and classification of types of schizophrenia or schizoaffective disorder, previous studies have adopted cut-off points. For instance, a review of adolescent research used the age of 17 to distinguish child- and adolescent-onset from adult-onset schizophrenia [26]. Because 17 years of age is the cut-off found in most studies and clinical settings [26], we selected 17 years as the cut-off for early-onset schizophrenia.

## Methods

### Participants

A total of 113 inpatients with a Diagnostic and Statistical Manual of Mental Disorders, Fourth Edition (DSM-IV) [12] diagnosis of schizophrenia (N = 59) or schizoaffective disorder (N = 54) recruited from among all patients hospitalised at one psychiatric service during the continuous one-year period of time selected for the study, were individually participated in the study. All patients who had experienced an illness duration of over one year were eligible for recruitment from the chronic inpatient unit of a general hospital, where they had to have been hospitalised for treatment of an acute psychotic exacerbation for at least 60 days before recruitment. Prior to commencing the study, ethical approval was obtained from the Institutional Review Board of Tri-service General Hospital, National Defense Medical Center in Taiwan. Following a comprehensive explanation of the study, subjects were asked to give their written informed consent. Of the 113 inpatients initially invited to participate in the study, 9 refused to participate, yielding a final sample of 104 subjects (92% of the initial sample). Subjects were between the ages of 19 and 60 years. Of the final sample, 52 patients were diagnosed with schizophrenia and 52 with schizoaffective disorder. Subsequent to the initial evaluation, participants also underwent a comprehensive screening and assessment. The clinical procedure used for this purpose involved the administration of a structured clinical interview, a detailed medical history review and physical examinations. Patients who had evidence of organic brain pathology including cerebral tumour, epilepsy, systemic disease, history of cranial trauma, brain surgery, or history of substance abuse or dependence in the past or present were excluded from this study. To obtain this information, the interviewer systemically inquired about the chronology of psychotic symptoms and the level of impairment they produced. Prior to entering the study, only 1% (n = 1) of the patients were taking typical or first-generation antipsychotics (Haloperidol), and 99% (n = 103) were taking atypical antipsychotics, including Risperidone (17%; n = 17); Quetiapine (21%; n = 22); Amisulpride (7%; n = 9); Aripiprazole (13%; n = 13); Ziprasidone (1%; n = 1); Zotepine (4%; n = 4); Olanzapine (5%; n = 5); and Clozapine (32%; n = 32). Finally, 18 patients (n = 18) had been prescribed antipsychotic depot medications (Haloperidol decanoate n = 15; and Risperidone Consta n = 3). The antipsychotic prescriptions for 85 subjects had been unchanged for at least three months prior to their recruitment, and only 19 subjects had undergone small changes in their prescriptions during the six months prior to recruitment. These 19 subjects were evaluated as clinically stable by the responsible psychiatrist.

Baseline demographic data consisted of gender, age, educational level, and body mass index (BMI). A semi-structured interview to determine the age of illness onset, the duration of illness, and recurrence of previous hospitalizations was obtained from the one responsible psychiatrist. Note that data were also extracted from all available information, including hospital records and information from family members. The age of illness onset was defined as the age when the patient met DSM-IV criteria [12] for the first time. The duration of the illness was defined as the time since the first psychotic episode.

### Measures

The following assessments were administered at the same time with reference to the respondent's behaviour and experience over the previous 12 months.

The Self-Appraisal of Illness Questionnaire (SAIQ) was used to assess attitudes toward schizophrenia among patients receiving psychiatric treatment. The SAIQ is an assessment tool developed for use in the clinical setting that was derived closely from the concept of the Patient's Experience of Hospitalization (PEH) questionnaire [27]. This scale is a self-report instrument composed of 17 items. Participants were asked to rate the extent to which they agreed with each statement by using a four-point Likert scale ranging from 0 (i.e., "do not agree at all") to 3 (i.e., "agree completely"). The internal consistency of the scale was 0.867 and the test-retest reliability was 0.82. It was concluded that the Need for Treatment and Presence/Outcome of Illness subscales could be used as brief screening instruments for clients with schizophrenia who may be at risk for treatment non-compliance due to a lack of insight into their illness [27]. Lower SAIQ subscale scores indicate less awareness of one's psychiatric illness.

The patients' global psychopathology was evaluated with the Positive and Negative Syndrome Scale (PANSS) [28]. The PANSS was developed in an attempt to provide a more comprehensive assessment of the symptoms of schizophrenia. It is widely used in clinical and research settings and is regarded as a reliable means of symptom assessment. In the present study, four analytically-derived PANSS components were used: positive component, negative component, cognitive component and total score [28].

Action without planning or reflection is central to most definitions of impulsivity. Prior research has demonstrated that impulsivity is a considerably complex behavioural construct [29]. To quantify impulsivity, the Barratt Impulsiveness Scale (BIS) was used as a self-report assessment [30]. This scale relies mainly on subjects' recall of behaviours or attitudes. It contains 30 items measuring three aspects of impulsivity. Attentional/cognitive impulsivity is a lack of cognitive persistence with an inability to tolerate cognitive complexity; motor impulsivity is a tendency to act impulsively; and non-planning impulsivity refers to a lack of sense of the future [30].

The Beck Depression Inventory (BDI) is a 21-item self-report scale [31]. Each item consists of four alternative statements that reflect gradations in the intensity of a particular depressive symptom (rated in severity from 0 to 3). The results are scored by summing the responses to each of the items to obtain a total depression score (range = 0-63). The psychometric properties of the inventory have been reviewed by Beck and Steer [31].

The Anxiety Checklist (ACL) was designed to assess the severity of anxiety symptoms in depressed patients [32]. This scale consists of 21 items that represent somatic, affective, and cognitive symptoms. The ACL has been shown to exhibit good internal consistency (alpha = 0.92) and test-retest reliability, r(58) = 0.75, over one week [32].

The Beck Hopelessness Scale (BHS) is a 20-item true-false self-report instrument that assesses the degree of pessimism exhibited by an individual [33]. Each of the 20 items is scored as either 0 or 1. The total score is the sum of the individual item scores (range = 0-20). In a sample of 294 hospitalised patients who had attempted suicide, the Kuder-Richarson reliability (KR21) coefficient for the Beck Hopelessness Scale was 0.93, and all of the item-total correlations, ranging from 0.39 to 0.76, were significant [33].

To assess patients' present suicidal risk, the Scale for Suicide Ideation (SSI), which includes 19 items that evaluate the severity of current suicidal ideations and wishes, was used [34]. It is based on clinical systemic observations and interviews with suicidal subjects. Each item is composed of three choices that range from 0 (least severe) to 2 (most severe). The total score is obtained by summing the item ratings yielding total scores between 0 and 38. These items assess the frequency and duration of suicidal thoughts as well as patients' attitudes towards them [34].

In this study, the comprehensive strategy for evaluating the patients' quality of life (QOL) involved two main domains: clinician-rated (objective) and self-rated (subjective) assessments. The objective evaluation of clinical course and social functioning of the patient was based on the Global Assessment of Functioning (GAF), which is a measure of overall psychological disturbance as rated by the clinician [12]. In addition, the Taiwanese version of the WHOQOL-BREF was used as the subjective aspect-specific scale in this study. Like the standard WHOQOL-BREF questionnaire, it defines four domains related to QOL (physical health, psychological health, social relationships, and environment) and measures the facets of QOL and general health [35,36]. It contains 28 items, including 26 standard items from the WHOQOL-BREF and two culturally relevant items [35,36]. The 26 standard items are comprised of one item from each of the 24 facets of the WHOQOL-100 and two items from the overall QOL and general health facet. In a study by Yao et al. [36], exploratory and confirmatory factor analyses of the Taiwanese version of the WHOQOL-BREF revealed a four-factor model (physical health, psychological health, social relationships and environment). Internal consistency (Cronbach's alpha) coefficients ranged from 0.7 to 0.77 for the four domains. Test-retest reliability coefficients with intervals of two to four weeks ranged from 0.41 to 0.79 at the item/facet level and 0.51-0.64 for inter-domain correlations (all p < 0.01). In the present study, the four domain scores (physical health, psychological health, social relationships and environment) were calculated by the standard scoring algorithms of the Taiwanese version of the WHOQOL-BREF. Scores ranged from 4 to 20. Additionally, the two items measuring overall quality of life (Facet G1: In general, how would you evaluate your quality of life?) and general health (Facet G4: In general, are you satisfied with your health?) were averaged to represent overall health-related QOL [35,36].

### Statistical analysis

All statistical tests were carried out using the Statistical Package for the Social Science (SPSS), version 15.0 for Windows.

Data analysis was conducted in three phases. Initially, comparisons of demographic and clinical variables between the schizophrenia and schizoaffective groups were conducted using independent samples t-tests and Mann-Whitney U-tests for continuous and categorical variables, respectively. Additionally, given the theoretical positions taken for the study as briefly reviewed above, two analytical approaches were applied in our study. Spearman's correlation coefficient was used to assess relationships between the age of illness onset and insight into illness, psychopathology, symptom rating scales, and QOL variables. For exploratory analysis of associations with clinical variables, the age of illness onset factor was dichotomised. We chose 17 years of age as the cut-off for early-onset schizophrenia based on a recent international consensus [26]. Subjects who were aged 17 or younger were considered early-onset, and subjects older than 17 years were considered adult-onset cases [26]. To identify predictive variables, binary logistic regression models were created using the forward stepwise method to identify clinical variables that were good predictors of age of illness onset. To assess the effects of age of onset separately from influences of current age and duration of illness, binary regression analysis was repeated after removing any significant independent predictors for which associations were simply due to the effects of current age and duration of illness. All variables that were significant (p < 0.01) or showed a trend toward significance (p < 0.05) in univariate analyses were included in the regression analyses (details of the included variables are presented in the results section). As our study was exploratory, we considered trends as well as significant findings. In this study, we decided to apply a stepwise regression because we needed to balance sensitivity and utility. We also identified enough predictors to be sufficiently sensitive to explain the patients' ages of illness onset, but few enough to avoid interaction effects that could result in utility problems [37]. The Wald test was used to examine the effects of the explanatory variables. Finally, the present study compared psychopathology and other clinical characteristics between early- and adult-onset illness. The comparison was made by splitting the patients into two groups based on age of onset, then using univariate two-way analysis of covariance (ANCOVA) to identify differences between early- and adult-onset patients in the remaining demographic and clinical variables. To control for the effects of current age and duration of illness, these factors were regarded as covariates in the ANCOVAs.

## Results

### Participants' characteristics and clinical evaluations

The average age of patients at the time of assessment was 39.24 years (standard deviation [SD] = 10.29), ranging from 19 to 60 years, and the mean duration of receiving education was 12.88 years (SD = 2.75), ranging from 9 to 18 years. Fifty-two (50%) of the patients were male and fifty-two (50%) were female. Marital statuses of the patients were as follows: 10 (10%) married, 78 (75%) unmarried, and 16 (15%) divorced or widowed. Fifty-six percent (n = 58) had a BMI in the normal range (less than 25), 29% (n = 30) were overweight, and 15% (n = 16) were obese (greater than 30). The mean age of illness onset was 24.14 years (SD = 7.58 years; range: 15-51 years); the mean illness duration was 15.1 years (SD = 8.56 years; range: 4-37); and patients had an average of 7.24 previous hospitalisations (SD = 4.28 years; range: 2-25).

A breakdown of demographic and clinical characteristics by diagnosis is presented in Table 1. The two groups were similar in terms of sex, current age, age of illness onset, illness duration, previous hospitalisations, and some clinical rating scales (all p-values > 0.05). Patients with a diagnosis of schizophrenia had significantly higher QOLs according to the total score and four domain scores, whereas patients with a diagnosis of schizoaffective disorder had higher SAIQ, PANSS, BDI, and BIS scores (all p-values < 0.05).

**Table 1:** Means (and SD) of demographic and clinical characteristics for the schizophrenia (n = 52) and schizoaffective (n = 52) groups

	Schizophrenic disorder Mean (SD)	Schizoaffective disorder Mean (SD)	t	Significance
**Age**	40.23 (10.06)	38.25 (10.51)	0.982	0.329
**Education**	12.88 (2.66)	12.88 (2.86)	0.000	1
**BMI**	24.95 (5.23)	25.27 (4.03)	-0.354	0.724
**Age of illness onset**	25.56 (7.94)	22.73 (6.98)	1.927	0.06
**Illness duration**	14.67 (8.85)	15.52 (8.33)	-0.502	0.617
**Previous hospitalization**	6.51 (3.80)	7.96 (4.64)	-1.734	0.086
**SAIQ need for treatment**	10.13 (2.90)	11.31 (3.25)	-1.941	0.055
**SAIQ presence/outcome of illness**	8.29 (3.27)	9.88 (3.78)	-2.303	0.023*
**PANSS positive**	15.13 (3.44)	15.48 (3.44)	-0.513	0.609
**PANSS negative**	19.25 (5.32)	19.11 (4.50)	0.139	0.889
**PANSS cognitive**	19.35 (5.16)	21.79 (4.25)	-2.633	0.01*
**PANSS total score**	73.71 (16.33)	80.38 (12.51)	-2.339	0.021*
**BIS motor**	14.67 (5.38)	16.63 (5.97)	-1.76	0.081
**BIS attention**	9.21 (4.52)	11.25 (4.68)	-2.26	0.026*
**BIS non-planning**	15.25 (5.53)	16.90 (4.81)	-1.628	0.107
**BIS total score**	39.13 (13.28)	44.79 (12.75)	-2.215	0.029*
**BDI**	12.25 (10.14)	16.87 (12.78)	-2.056	0.042*
**ACL**	14.52 (13.7)	17.79 (15.47)	-1.115	0.252
**BHS**	6.42 (4.23)	6.52 (4.32)	-0.115	0.909
**SSI**	3.56 (5.33)	4.71 (7.16)	-0.932	0.353
**QOL G1F**	3.29 (0.8)	2.88 (0.81)	2.56	0.012*
**QOL G4F**	3.29 (0.91)	2.58 (1.07)	3.64	<0.001**
**QOL physical**	23.10 (4.53)	21 (3.71)	2.58	0.011*
**QOL psychological**	19.0 (4.14)	16.27 (4.12)	3.37	0.001**
**QOL social**	12.98 (4.35)	10.88 (3.01)	2.86	0.005**
**QOL environmental**	26.60 (6.33)	25.79 (4.58)	0.745	0.458
**QOL total score**	81.67 (13.99)	73.94 (11.75)	3.05	0.003**
**GAF**	52.01 (13.0)	50.67 (10.0)	0.592	0.555

*p < 0.05; **p < 0.01

Abbreviations: SAIQ = Self-Appraisal of Illness Questionnaire; PANSS = Positive and Negative Syndrome Scale; BIS = Barrett Impulsiveness Scale; BDI = Beck Depression Inventory; ACL = Anxiety Checklist; BHS = Beck Hopelessness Scale; SSI = Scale for Suicide Ideation; QOL = Quality of Life; GAF = Global Assessment of Functioning.

### Correlation and regression analyses of age at illness onset

To evaluate the relationship between age of illness onset and these clinical characteristics, a series of correlational analyses (Spearman's rho) was conducted. The age of onset for all subjects was found to be significantly correlated with insight into outcome/presence of illness (Spearman's rho = -0.21, p < 0.05), PANSS components, except for the positive component, and total scores (Spearman's rho = -0.199 to -0.257, p < 0.01), BIS total and subscale scores (Spearman's rho = -0.266 to -0.354, p < 0.01), and QOL total and domain scores, with the exception of the environmental domain (Spearman's rho = 0.248 to 0.336, p < 0.01), but it was not correlated with sex, education, previous hospitalisations, BDI, ACL, BHS, SSI or GAF. Because a large number of correlation factors were examined in this analysis, the threshold for significance was set at p < 0.05.

In this study, 19 patients were early-onset cases. Using binary regression (Table 2), we examined the relationship of candidate predictors to age of onset in models including sex, education, total number of previous hospitalisations, two SAIQ scores that assessed global insight into illness, and scores on the PANSS, BIS, BDI, ACL, BHS, SSI, QOL, and GAF as independent variables and the current age and duration of illness as a priori confounding independent variables. The analysis resulted in two models predictive of age of onset. The first model (-2 log likelihood = 88.27, χ^2 ^= 10.63, p < 0.001) contained only one predictor: cognitive impairment (odds ratio, OR = 0.732; 95% confidence interval, CI = 0.62-0.864). The second model (-2 log likelihood = 83.32,χ^2 ^= 15.58, p = 0.015) contained two predictors: impulsivity traits (OR = 0.94; 95% CI = 0.447-0.744) and cognitive impairment (OR = 0.58; 95% CI = 0.872-0.985). We found that both cognitive impairment and impulsivity traits, as rated with the PANSS and BIS, respectively, were inversely related to age of onset in this sample of stabilised, schizophrenia spectrum disorders.

**Table 2:** Multivariate logistic regressions (stepwise) with age of illness onset as the dependent variable

Predictors	Beta	SE	Wald	Significance	OR	95% CI
Step 1						
Cognitive	-0.312	0.085	13.538	<0.001	0.73	0.62-0.864
Constant	8.488	2.023	17.606	<0.001	0.057	
Step 2						
Impulsivity	-0.076	0.031	6.065	0.015	0.94	0.872-0.985
Cognitive	-0.551	0.13	17.948	<0.001	0.58	0.447-0.744
Constant	6.824	2.235	9.325	0.002	0.021	

Abbreviations: Cognitive = cognitive component of the Positive and Negative Syndrome Scale; Impulsivity = total score of Barratt Impulsiveness Scale; OR = Odds ratio; CI = Confidence Interval.

### Mean test scores for age of onset groups (ANCOVAs)

Mean test scores for early- and adult-onset patients are presented in Table 3. After adjusting for current age and illness duration, early- and adult-onset patients had significantly different clinical characteristics. No differences were found in education, previous hospitalisations, PANSS positive component, SAIQ, several symptom ratings, and QOL, with the exception of the psychological domain, between early- and adult-onset patients. As presented in Table 3, early-onset patients had significantly higher scores on the negative and cognitive domains and total score on the PANSS as well as the BIS attentional and non-planning subscales and total score. However, the mean score in the psychological domain of QOL in early-onset patients was lower than that in the group of adult-onset patients (all p-values < 0.05).

**Table 3:** Means (and SD) test scores for early-onset and adult-onset schizophrenia spectrum disorders and the results of an ANCOVA with current age and duration of illness as covariates

	Early onset (n = 19)		Adult onset (n = 85)			
	Mean	SD	Mean	SD	F(df = 103)	Significance
**Education level**^**a**^	12.0	2.00	13.38	2.86	0.052	0.821
**BMI**	26.28	6.29	24.85	4.20	2.373	0.127
**Previous hospitalisations**	7.37	3.93	7.21	4.38	0.004	0.95
**SAIQ need treatment**	10.63	3.34	10.74	3.09	0.069	0.793
**SAIQ presence/outcome**	9.47	3.52	9.00	3.64	0.308	0.58
**PANSS positive**	15.26	3.45	15.32	3.45	0.000	0.988
**PANSS negative**^**a**^	22.84	4.35	18.36	4.66	13.134	<0.001**
**PANSS cognitive**^**a**^	24.63	4.07	19.66	4.57	14.919	<0.001**
**PANSS total score**^a^	86.37	13.63	74.96	14.38	6.236	0.014*
**BIS motor**^**a**^	18.79	5.99	14.95	5.48	1.114	0.294
**BIS attentional**^**a**^	13.11	4.83	9.59	4.44	3.974	0.049*
**BIS non-planning**^**a**^	19.58	4.06	15.29	5.15	3.998	0.047*
**BIS total score **^**a**^	51.47	12.32	39.84	12.57	3.938	0.05*
**BDI**	17.89	13.89	13.79	11.13	0.092	0.763
**BAI**	19.00	16.01	15.52	14.14	0.07	0.792
**BHS**	7.21	4.5	6.31	4.21	0.414	0.646
**SSI**	3.68	6.90	4.24	6.21	0.777	0.38
**QOL G1F**^**a**^	2.68	1.00	3.18	0.76	1.035	0.312
**QOL G4F**^**a**^	2.26	0.93	3.08	1.03	2.707	0.103
**QOL physical**	20.52	4.41	22.39	4.17	0.233	0.063
**QOL psychological**^**a**^	15.05	4.93	18.21	4.0	3.936	0.05*
**QOL social**^**a**^	10.32	3.61	12.29	3.85	0.403	0.527
**QOL environmental**	25.58	5.88	26.33	5.46	0.001	0.971
**QOL total score**^**a**^	71.47	15.04	80.23	12.71	0.826.	0.366
**GAF**	47.63	8.72	52.18	11.99	1.895	0.172

*p < 0.05; **p < 0.01 ^**a **^Indicates significant differences in paired t-test

Abbreviations: SAIQ = Self-Appraisal of Illness Questionnaire; PANSS = Positive and Negative Syndrome Scale; BIS = Barrett Impulsiveness Scale; BDI = Beck Depression Inventory; ACL = Anxiety Checklist; BHS = Beck Hopelessness Scale; SSI = Scale for Suicide Ideation; QOL = Quality of Life; GAF = Global Assessment of Functioning.

## Discussion

Among the numerous clinical characteristics used to clarify the schizophrenia spectrum disorders, the age of illness onset is widely accepted as having particularly powerful clinical and prognostic significance. The complexity and variety of effects of the age of onset in schizophrenia patients reported in the literature are due not only to the difficulty in operationally defining the age of illness onset but also to the broad distribution of ages of onset from preadolescence to later adulthood. In this cross-sectional study, there was evidence for statistically significant relationships between age of onset and cognitive impairments and impulsivity traits in this group of schizophrenia spectrum disorders. Patients with early onset had higher levels of cognitive impairments and impulsivity traits than did patients with adult onset. This is compatible with the generally accepted view of early-onset cases as having unique clinical and prognostic consequences. However, we do not have any evidence for a causal relationship between age of onset and cognitive impairments and impulsivity traits. No definitive conclusion can be drawn until longitudinal prospective studies are carried out.

The mean age of onset for all schizophrenia patients who participated in this study was slightly older than is generally reported in populations of schizophrenia patients [38], particularly when recorded as the year of life when the subject first met DSM-IV [12] criteria. A possible explanation is that a large proportion of our patients (about 70%) presented with the paranoid type of schizophrenia, which is characterised by a considerably older age of onset (mean age of 28.5 years vs. 19.9 years in patients with non-paranoid schizophrenia). The present results also show that schizophrenia patients were not different from patients with schizoaffective disorder in terms of age of onset. There is a growing body of research specifically regarding early-onset schizophrenia [16-18], but research regarding youths with schizoaffective disorder is sparse [39]. In fact, most studies include schizoaffective disorder as an exclusionary criterion or combine both diagnoses into one group for data analysis [39]. Further complicating matters is the fact that these diagnoses are often contingent on a longitudinal illness course, yet diagnosis is generally made using cross-sectional information. The DSM-IV diagnostic criteria for schizoaffective disorder require that mood episodes be present for a substantial portion of the duration of the illness [12]. This diagnostic assignment may change over time as the course and presentation of psychotic symptoms become obvious [39,40]. For example, in the clinical setting, a patient's diagnosis can change from schizophrenia at baseline to schizoaffective disorder at discharge [40]. Further research will be needed to help clinicians distinguish schizophrenia from schizoaffective disorder in patients with early-onset schizophrenia. Despite this, one of the most salient and overarching findings of our study is that the patients with schizophrenia and schizoaffective disorder are more similar than different in terms of their demographic and symptom profiles. Our findings provide additional support for shared etiological and pathophysiological features across schizophrenic disorder groups. Such information will have important prognostic and treatment implications.

Researchers have shown that age of onset may not necessarily act as a unique determinant in the course of schizophrenic disorder as evidence indicates that men have an earlier age of illness onset than women [3-6] and a more severe course of illness, particularly in the short and medium terms [41]. A remarkable finding in the present study was that we were not able to establish differences between male and female patients in demographic variables, including age at onset and symptom severity, or in total scale or subscale scores. This is in contrast with previous studies that found symptomatic differences between the sexes. In these previous studies, negative symptoms were consistently found to be more severe in men [42]. This discrepancy might be due not only to differences in the rating scales applied but also to sample differences. The lack of evidence for a sex difference is difficult to explain. Our patients' mean age (mean age = 40.57 years) was higher than those of other studies [43], the female patients (mean age = 41 years) were older than the males (mean age = 40 years), and female patients (mean duration = 15.25 years) had a longer illness duration than males (mean duration = 14.94 years); this might be related to a putative progressive reduction in symptom differences. The results indicate that the differences in clinical characteristics of schizophrenic disorders between early- and adult-onset patients may be more pronounced than those between patients of different sexes, but an interaction effect might be present between sex and age of onset.

A number of independent cognitive deficits were apparent in our chronic schizophrenic patients, especially in early-onset cases. The findings of the current study and the study of Hoff et al. (1992) indicate a more generalised, diffuse cognitive deficit in chronic schizophrenic disorders [44]. Our results also support the assertion of the DSM-IV (1994) that schizophrenia patients with younger ages of onset are more cognitively impaired [12]. It seems that an earlier onset of schizophrenia is associated with a more severe course irrespective of duration of illness [45]. Given its cross-sectional nature in this study, however, no conclusion can be drawn regarding causality and alterative explanations of the findings cannot be ruled out. For instance, it is possible that patients with adult onset had better response to antipsychotic medications, thereby reducing their severity of symptoms. Specifically, we used the cognitive component of the PANSS to assess cognitive function in patients with schizophrenia. It has been documented that higher scores on the PANSS cognitive component are significantly correlated with poorer performance on neuropsychological tests [46].

Action without planning or reflection is central to most definitions of impulsivity. In the present study, we used the BIS questionnaire, which tends to measure impulsivity as a stable characteristic, as a self-reported assessment of impulsivity [30]. There were significant associations between early age of onset and severity of impulsivity traits. Previous reports have suggested that schizophrenic patients are likely to exhibit impairments on a wide range of neuropsychological tasks, including attention and executive functioning [47]. Heaton et al. (2001) showed that the neuropsychological impairment in patients with schizophrenia appeared to remain stable regardless of baseline characteristics and changes in clinical state [48]. Reduced P300 amplitude, a neurophysiological parameter associated with impulsivity and behaviour disinhibition [49], and a P300 effect size (d) that was smaller in amplitude and longer in latency have been observed in schizophrenic patients compared to normal controls, with the strongest effects obtained from the auditory oddball task [50]. Therefore, it is plausible that psychopathological and neurocognitive impairments in schizophrenic patients are mediator variables responsible for the effect of impulsivity on the age of onset in schizophrenia spectrum disorders. The results reported in the present study support this relationship. Because impulsivity is present as a relatively stable trait, it would appear that greater impulsivity is already present at onset in early-onset schizophrenia. However, no definitive conclusion can be drawn until further prospective studies are performed.

Compared with the regression model, significant effects of age at onset were found in the negative symptom component, cognitive component, and the total score, but not in the positive component of the PANSS in the ANCOVAs. Patients with early illness onset scored higher on negative symptoms, cognitive symptoms, and general psychopathology than did patients with adult-onset illness. To further evaluate the magnitude of the predicted difference, an effect size test was conducted. The standardised effect size difference for cognitive impairment between groups was 0.387, reflecting a medium-sized effect [51]. Moreover, the standard effect sizes for negative symptoms and impulsivity traits were 0.427 and 0.511, respectively, also reflecting medium-sized effects [51]. However, the standardised effect size difference for positive symptoms between groups was 0.121, reflecting a smaller effect size [51]. These results agree with those of some previous systemic studies [52]. Similarly, some studies have reported that negative thought disorder was less severe in patients with older ages of illness onset. However, there was no effect of age of onset on the depressive symptoms in this study, a finding that is consistent with other comprehensive studies [4,52]. Together, given the exploratory nature of these studies, these data suggest that any phenomenon related to age of onset in schizophrenia based on these preliminary findings should be treated with caution.

In the present study, considering the results of t-tests for differences between the early-onset and adult-onset groups, it was expected that education would be related to age of onset as patients should have completed less schooling if their first episode occurred when they were still attending school. A likely explanation is that our patients with earlier ages of onset had poor educations due to the cognitive dysfunction associated with poorer outcomes in early-onset schizophrenia [12,53]. However, this significant effect was appreciably reduced after controlling for illness duration and current age. The findings suggest that the difference in the educational levels between the two groups may be strongly affected by illness duration and current age. It is difficult to estimate the confounding influence of illness duration on our test results due to the retrospective design.

It is uncertain, however, whether the effects of age of onset found in the present study reflect qualitatively specific schizophrenia or merely quantitative differences in psychopathology and impulsivity between early- and adult-onset illness in our patients. One recent study reported that the relationship between an older age of onset and less severe negative symptoms is also present in chronically ill schizophrenic patients with an age of onset of younger than 45 years [54]. Thus, future research is needed in this area, particularly concerning the potential consequences of age of onset, utilising different clinical measures (especially as the results indicate that early onset is a risk factor) and a broader array of measures tor precisely define the course of schizophrenic disorders.

In stabilised schizophrenic patients, assessment of subjective QOL has good reliability and concurrent validity [55,56]. Hence, measurement of subjective QOL may be considered as a pertinent indictor of the state of health of stabilised schizophrenic patients [56]. The present study tested the relationship of age of onset with the QOL of patients with schizophrenia spectrum disorders by using t-tests. The results showed that patients with an early onset of schizophrenia spectrum disorders were likely to have worse QOLs than those with an adult illness onset. A partial explanation for this may lie in the fact that an early onset of illness has been found to be a predictor of unfavourable prognosis and is correlated with higher global severity [57], higher rates of chronicity, and more probable impairments in cognitive performance [58]. However, this significant effect was largely reduced after controlling for illness duration. The findings suggest that the difference in QOL levels between the two groups may be strongly affected by illness duration. Besides, in patients with schizophrenia, adaptation and significant improvement in subjective QOL may be assumed to occur at a later stage of illness [59]. This finding is compatible with the results of our study, which showed that older patients are more satisfied with their lives than younger patients are. (Pearson's r = 0.218, p < 0.01).

There are some limitations of our research. First, only inpatients in the chronic setting were recruited in the present study. The results could not demonstrate whether the effect of age of onset as measured in our study indicates a trait or state characteristic. Additionally, we could not generalise our findings to all schizophrenia subjects. Thus, replication of the current findings in stabilised outpatients will be necessary. Second, because the present study required informed consent and included psychopathological assessments, we did not include subjects who were very uncooperative. Thus, we lack demographic characteristics of non-volunteers. However, it should be noted that those uncooperative subjects were demographically different from the volunteers, and thus the influence of our results might be limited. Third, as noted above, the size of the early-onset group was relatively small, which likely limited our ability to detect group differences due to low statistical power, but this may reflect a greater prevalence of adult-onset schizophrenic disorder cases [16]. Fourth, it is important to emphasise that methodological problems such as the retrospective design limit our interpretation. All data on the illness course, however, were based on information documented at the time of inpatient treatment, including the age of onset of the first psychotic episode and other demographic and clinical characteristics, which can be biased by recall effects. Thus, a prospective comparison of characteristics at illness onset of patients with schizophrenia spectrum disorders will be necessary for future research. Finally, given the retrospective design of our study, the psychopharmacological variables were not controlled a priori, and thus it was not possible to determine the effects of the medications on some aspects of cognition and the clinical course of the disease.

## Conclusions

In summary, the present study showed that the variance of demographic and clinical characteristics in schizophrenia spectrum disorders may be greater between early- and adult-onset patients than between patients of different sexes. The findings are roughly in line with Howard's (2000) results [60]. Our statistical analyses also demonstrated that these significant associations were not accounted for by the duration of illness. Thus, the ability to recognise psychopathological symptoms of schizophrenia to start psychopharmacological and psychosocial interventions as soon as possible is becoming a central issue in clinical practice [61]. Accordingly, further investigation is needed to gain a better understanding of the age effect on the illness process of schizophrenia spectrum disorders.

## Competing interests

The authors declare that they have no competing interests.

## Authors' contributions

YCK wrote draft of the manuscript. YCK and YPL conceptualized and designed the study. YCK collected and analyzed the data. YPL supervised the study. YCK analyzed the data further and wrote the final manuscript. YPL helped to draft and revised the manuscript. All authors read and approved the paper.

## Pre-publication history

The pre-publication history for this paper can be accessed here:

http://www.biomedcentral.com/1471-244X/10/63/prepub
